# Targeting the ERK Signaling Pathway in Melanoma

**DOI:** 10.3390/ijms20061483

**Published:** 2019-03-25

**Authors:** Paola Savoia, Paolo Fava, Filippo Casoni, Ottavio Cremona

**Affiliations:** 1 Department of Health Science, University of Eastern Piedmont, via Solaroli 17, 28100 Novara, Italy; 2 Section of Dermatology, Department of Medical Science, University of Turin, 10124 Turin, Italy; fava_paolo@yahoo.it; 3 San Raffaele Scientific Institute, Division of Neuroscience, via Olgettina 58, 20132 Milano, Italy; casoni.filippo@hsr.it; 4 Università Vita Salute San Raffaele, via Olgettina 58, 20132 Milano, Italy

**Keywords:** ERK pathway, target therapy, metastatic melanoma, BRAF inhibitors, MEK inhibitors, ERK inhibitors, NRAS inhibitors, melanoma treatment

## Abstract

The discovery of the role of the RAS/RAF/MEK/ERK pathway in melanomagenesis and its progression have opened a new era in the treatment of this tumor. Vemurafenib was the first specific kinase inhibitor approved for therapy of advanced melanomas harboring BRAF-activating mutations, followed by dabrafenib and encorafenib. However, despite the excellent results of first-generation kinase inhibitors in terms of response rate, the average duration of the response was short, due to the onset of genetic and epigenetic resistance mechanisms. The combination therapy with MEK inhibitors is an excellent strategy to circumvent drug resistance, with the additional advantage of reducing side effects due to the paradoxical reactivation of the MAPK pathway. The recent development of RAS and extracellular signal-related kinases (ERK) inhibitors promises to add new players for the ultimate suppression of this signaling pathway and the control of pathway-related drug resistance. In this review, we analyze the pharmacological, preclinical, and clinical trial data of the various MAPK pathway inhibitors, with a keen interest for their clinical applicability in the management of advanced melanoma.

## 1. Introduction

Epidemiological estimates for melanoma identify it as the 20th most common cancer worldwide, with 287,723 of expected new cases in 2018, growing up to 301,694 in 2020, and 466,914 in 2040; it has an overall age-standardized incidence of 3.1/100,000 and a prevalence of 12.7/100,000, with more than 60,000 expected deaths/year (source: GLOBOCAN 2018, http://gco.iarc.fr/) (accessed on 1 March 2019) [1]. Melanoma is the fourth more commonly diagnosed cancer in Oceania, the sixth in Europe, and the seventh in North America, while it is a rather uncommon occurrence in Africa and Asia. In contrast, the melanoma mortality rate among all cancer types is at the 10th position in Oceania and the 17th position in Europe and North America. Although melanoma represents less than 4% of skin cancers diagnosed in the US, it accounts for more than 30% of skin cancer-related deaths (source: https://www.skincancer.org). The most important (if not, the only) non-genetic risk factor for melanoma is the ultraviolet (UV) radiation that escapes melanin absorption, leading to genetic changes as a result of two actions: (i) UVA-generated reactive oxygen species (ROS) (reviewed in References [2,3]) and/or (ii) direct UVB-driven DNA mutations/breaks (reviewed in References [4,5]). Meta-analyses of observational studies on melanoma found that especially intermittent sun exposure and sunburn history play a key role in melanomagenesis, whereas chronic occupational sun exposure is generally protective for this tumor unless associated with a poorly pigmented skin phenotype [6,7].

Notably, in these years, we are experiencing a continuous decrease in the melanoma mortality rate; this positive trend strikingly correlates with the introduction of kinase inhibitors in the treatment of this neoplasia in 2011. Combined therapies with different kinase inhibitors with or without immunological checkpoint inhibitors will possibly lead to a much better control of the disease with a further decrease in the mortality rate of this tumor, a key step toward the transformation of advanced melanoma from a rapidly fatal disease into a chronic condition.

## 2. Overview of Histopathology and Genetics of Melanoma

Melanoma, as other malignancies, arises and progresses through the accumulation of genetic mutations that lead to uncontrolled cell proliferation and the appearance of an invasive cell phenotype. 

In most cases, cutaneous melanomagenesis has the histopathological features of a linear stepwise process. It initiates with distinct precursor lesions (in the form of benign melanocytic nevi and/or intermediate lesions, also termed as dysplastic nevi) and, then, it proceeds through a series of increasingly malignant lesions (from melanoma in radial growth phase (including melanoma in situ), to melanoma in vertical growth phase (also termed as tumorigenic or invasive melanoma), and, finally, to metastatic melanoma with different degrees of aggressiveness) [8,9,10,11,12]. It is important to note that not all melanomas pass through these individual steps, with a possible tumor development direct from transformed melanocytes (reviewed in Reference [13]). The precise sequence of genetic alterations that drives melanocyte transformation is still incompletely understood but starts to be unveiled through the extensive application of next-generation sequencing (NGS) to the genetic characterization of precursor and melanoma lesions (reviewed in References [14,15,16,17]). 

Morphologically, a nevus (or mole) is a proliferation of non-dendritic, round/compact melanocytes, organized in clusters or nests of cells, with a peculiar tendency to retain pigment, and sometimes to migrate from the epidermis to the superficial dermis. Based on the location of melanocytes, nevi are classified into junctional, dermal, and compound, according to the presence of melanocytic clusters within epidermis, dermis, or both, respectively [12]. Approximately 30% of melanomas are associated with a nevus [18], although the malignant transformation rate of this latter lesion is a rare event, with a lifetime risk of any selected nevus to transform into melanoma ranging from 1:3164 for men to 1:10,800 for women [19]. Genetically, more than 80% of benign moles carry an upregulation of the MAPK pathway as a result of a single somatic mutation, i.e., the V600E mutation of the BRAF kinase (*BRAF^V600E^*) [9,20,21,22]; these *BRAF^V600E^* nevi have key histopathological features, being predominantly dermal, with melanocytes among collagen fibers, and with (peri)adnexal and perivascular growth [9,20,21,22].

A dysplastic nevus is an intermediate lesion, with a mixture of benign and malignant histological features, including the appearance of scattered cytological atypia, often associated with signs of inflammatory infiltration; this intermediate lesion may arise within a preexisting nevus or into a new site [8,12,13]. Although the definition and even the existence of the dysplastic nevus are still debated [10,11,23,24,25], the World Health Organization has set a general consensus for its morphological identification in two primary criteria and, at least, two accompanying minor criteria [26], which are widely followed [12]. Primary criteria are (i) proliferation of atypical nevomelanocytes in the basal epidermal layer (extending at least to three rete ridges or “pegs” beyond any dermal nevocellular component), and (ii) organization of this proliferation in a lentiginous or epithelioid-cell pattern [26]. Minor criteria are (i) the presence of lamellar fibrosis or concentric eosinophilic fibrosis, (ii) neovascularization, (iii) inflammatory response, and (iv) fusion of rete ridges [26]. Dysplastic nevi have a broader spectrum of driving mutations than benign nevi—while most of the intermediate lesions display the *BRAF^V600E^* mutation [9,27], there is a subset of dysplastic nevi harboring different MAPK pathway activating mutations, including *NRAS^Q61K^*^/*R*^ and two other BRAF pathogenic mutations, i.e., *BRAF^V600K^* and *BRAF^K601E^* [9]. In addition, dysplastic nevi harbor frequent activating mutations of the telomerase reverse transcriptase (TERT) promoter [9]. Moreover, dysplastic nevi have a higher mutational burden than benign moles [9] but lower than melanomas [27], and accumulate unique, albeit limited, UV-signature mutations, in the form of a specific pattern of cytosine-to-thymine mutation transitions [27].

Radial growth melanomas comprise melanomas in situ (i.e., melanomas whose cells are within the epidermis) and the microinvasive melanomas (i.e., melanomas that extend across the basement lamina in the upper dermis but without evidence of expansive growth or mitotic activity); these latter tumors include the superficial spreading melanoma, the acral lentiginous melanoma, and the *Lentigo Maligna* [12,13]. In radial growth melanomas, melanocytic cells cannot grow and proliferate outside the epidermis, in soft agar, or when implanted in nude mice [13], hence the alternative name of “non-tumorigenic” melanomas. Besides activating mutations of the MAPK pathway, radial growth melanomas are more consistently acquiring activating mutations in the TERT promoter (about 80% of the cases) in respect with dysplastic nevi [9,28]. However, despite increased TERT activity, telomeres are significantly shorter in these tumors than in the adjacent nevus, indicating that increased TERT activity in melanoma does not counteract or reverse telomere shortening produced by the high mitotic rate but possibly promotes genomic instability, which favors the genetic evolution and immortalization of cultured cells [29].

Vertical growth melanomas are characterized by the appearance of a nodular outgrowth [8,12,13]. Histologically, these tumors are distinguished by mitotically active nests of melanoma cells, which extends toward the papillary dermis, expanding eventually into the reticular dermis and the hypodermis [8,12,13]; their melanocytes can grow in soft agar and form tumors when implanted in nude mice (reviewed in Reference [13]). Genetically, vertical growth and metastatic melanomas show a further increase in TERT mutational rate and, more generally, in the mutational load over radial growth melanomas. Furthermore, advanced melanomas show the additional contribution of other activating mutations—including RAC1P29S [30,31], ribosomal protein S27 (RPS27) [32], and STK19 [33]—and the biallelic inactivation of a number of tumor suppressor genes—including the Glutamate Receptor Ionotropic, N-Methyl D-Aspartate 2A (GRIN2A, which is mutated in 30% of advanced melanomas) [34,35], the Cyclin-Dependent KiNase Inhibitor 2A (CDKN2A) [9,36], the Phosphatase and TENsin homolog (PTEN) [9,37], and the Tumor Protein 53 (TP53) [9,12,36]; melanocytes from advanced melanomas can grow in soft agar and form tumors when implanted in nude mice (reviewed in Reference [13]). 

The high mutation rate in melanoma is primarily attributed to the mutagenic effect of UV radiation (reviewed in Reference [7]), with two distinct pathways—a nevus-prone pathway promoted by intermittent sun exposure and sunburns, and a chronic sun exposure pathway, restricted to sun-sensitive people who progressively accumulate UV-related DNA damage to the sites of future melanomas (reviewed in Reference [38]). For a detailed description of the mutational landscape of the various types of melanomas, we refer the reader to specific reviews [16,17,39]. 

Besides gene mutations, heritable alterations in gene expression without a change in DNA sequence, i.e., epigenetic events, have a key role in melanomagenesis and its progression. Recent reviews have detailed these epigenetic alterations—methylation/demethylation of specific genes, non-coding RNAs including long non-coding RNAs and miRNAs, histone post-translational modifications including variant histones, and chromatin remodeling by specific complexes as the polycomb-repressive complex PRC2 [40,41,42]. 

In the next sections of the review, we will focus on the MAPK/ERK signaling pathway and its subversion in melanoma. 

## 3. The MAPK/ERK Signaling Pathway in Melanoma

The MAPK signaling pathways are evolutionarily conserved signal transduction pathways, which regulate a variety of fundamental cellular processes, including cell proliferation, differentiation, senescence, survival, transformation, and migration (reviewed in References [43,44,45]). These signaling pathways are characterized by the distinctive feature of linear/vectorial cascades of phosphorylation events, which spread from the cell membrane to the nucleus. The MAPK pathway activates with the interaction of a broad spectrum of extracellular signals—including mitogens, growth factors, and cytokines—with specific plasma membrane receptors. This interaction triggers a hierarchical cascade of phosphorylation events that ultimately terminates with the activation of specific MAP kinases (MAPKs)—their catalytic activity determines the specific signaling response of a given pathway, i.e., the activation/repression of a specific set of genes in the nucleus together with the activation/repression of specific cellular functions in the cytoplasm. In the mammalian genome, there are 14 MAPKs, which are organized into three groups, corresponding to as many distinct phosphorylation cascades and signaling pathways: (i) The extracellular signal-related kinases (ERKs), (ii) the c-Jun N-terminal kinases (JNKs), and (iii) the stress-activated protein kinases (p38/SAPKs) (reviewed in Reference [46]). Each group of MAPKs is activated, upon phosphorylation, by group-specific MAPK kinases (MAPKKs or MAP2Ks), which, in turn, are phosphorylated by group-specific MAPKK kinases (MAPKKKs or MAP3Ks), which are typically activated by group-specific small GTPases, upon binding to plasmalemma receptors. While the MAPK/JNK and the MAPK/p38 signaling pathways are mostly related to the regulation of the immune response and are activated by cellular stress conditions (including the oxidative, genotoxic, and osmotic stress), the MAP/ERK signaling pathway plays a crucial role in cancer development by promoting cell proliferation and migration; this latter pathway is mainly activated by growth factors (reviewed in References [47,48]). 

The MAPK/ERK signaling is essential for melanoma development and progression—the mutational landscape outlined above shows that the most frequent driving mutations in melanomagenesis are activating mutations of this pathway; moreover, the reactivation of the MAPK signaling by genetic and epigenetic events is the principal mechanism for acquired resistance to target therapy in this tumor (see below). The MAPK/ERK signaling typically starts with the activation of a growth factor/cytokine receptor tyrosine kinase (RTKs), which, upon ligand binding, dimerizes and autophosphorylates, generating multiple docking sites for adaptors (mainly Grb2) that link guanine nucleotide exchange factors (GEFs, mainly Sos) to RTKs (reviewed in References [49,50,51]). GEFs catalyze the dissociation of GDP from Ras-GTPases, thus favoring the conversion of the inactive Ras-GDP to the functionally active Ras-GTP at the plasma membrane; the opposite reaction is catalyzed by GTPase activating proteins (GAPs), which switches off Ras by boosting its GTPase activity (reviewed in References [49,51,52]); GDIs are a further regulatory layer of RAS GTPases (reviewed in References [49,53]). The Ras family of small GTPases is composed of 39 members [54] of which only three are frequently mutated in solid tumors—*KRAS* (accounting for about 85% of all *RAS* mutations), *NRAS* (about 15%), and *HRAS* (<1%) (reviewed in Reference [55]). These Ras-GTPases favor the aggregation of specific members of the MAP3K family—namely, ARaf, BRaf, and CRaf—to form kinase-active homodimers or heterodimers (reviewed in Reference [56]); based on the mutational status and potency in MEK activation, BRaf is the key MAP3K in melanomagenesis, followed by CRaf [57]. The Raf dimers are phosphorylating two specific MEKs among the seven MEK genes present in the mammalian genome—i.e., MAP/ERK Kinase 1 (MEK1/MAP2K1) and MEK2/MAP2K2—at specific serine residues (reviewed in References [58,59,60]). MEK1/2 proteins, in turn, catalyze a dual phosphorylation on threonine and tyrosine residues of a specific tripeptide sequence (Thr-Glu-Tyr) of the Extracellular signal-Regulated protein Kinase 1 (ERK1/MAPK1) and ERK2/MAPK2 (reviewed in References [46,61]); dephosphorylation by dual specificity phosphatases (DUSPs) modulates ERK activity, with DUSP5 inhibiting ERKs in the nucleus [62] and DUSP6 in the cytoplasm [63], establishing a negative feedback loop to prevent overactivation of signaling outputs [64]. In sum, the linearity of the MAPK phosphorylation pathway is granted by two consecutive phosphorylation steps with exclusive substrates, i.e., the Raf-mediated phosphorylation of MEK1/2, which in turn can only phosphorylate ERK1/2. 

While the proximal phosphorylation steps in the MAPK pathway are very restricted in terms of substrates, ERK1/2 activity results in a spread of serine/threonine phosphorylation to a large number of primary targets, both in the cytoplasm and in the nucleus, with a striking prevalence of the first subcellular location ([65] and related website: http://sys-bio.net/erk_targets). In the cytoplasm, where the vast majority of direct ERK targets are found, ERKs phosphorylate cytoskeletal and adherens junction components, thus promoting cell detachment from the extracellular matrix and motility. In the nucleus, where most of the indirect ERK targets are located, ERKs modulate the activity of proteins implicated in RNA transport/metabolism and of a number of transcription factors, including c-Fos, c-Myc, and c-Jun. Other common (direct and indirect) ERK targets include proteins implicated in cell cycle regulation, in apoptosis, and in many signaling pathways—including the TGF-beta, PI3K/AKT, Ras, MAPK, ErbB, insulin, and FoxO signaling pathways—leading to cross-talk and feedback regulation [65]. 

In normal mammalian cells, RAF kinases (ARaf, BRaf, and CRaf) are the most important RAS effectors [51]; additional major RAS effectors are the PI3K/AKT/PTEN pathway (reviewed in References [66,67,68]) and the Ral/RalGDS pathway (reviewed in References [69,70]). Not surprisingly, PI3K and Ral pathways are frequently upregulated in melanoma where they participate in the development of acquired drug resistance, frequently undermining BRAF/MEK target therapies in this neoplasia (reviewed in References [71,72,73] and see below). Furthermore, they crosstalk each other and with the MAPK pathway to a large extent, thus concurring to set several phenotypic traits of malignant transformation in melanoma (reviewed in References [71,74]). After the MAPK pathway, the PI3K/AKT pathway is the next most investigated signaling pathway in melanomagenesis. Its constitutive activation in this neoplasia typically results from increased expression of RTK ligands or mutations in the genes encoding for their cognate receptors (including MET, EGFR, PDGFRβ, IGF1R, and KIT (reviewed in References [75,76,77,78]), and/or, more frequently, in PI3K/AKT/PTEN/mTOR pathway genes (reviewed in References [75,79,80,81]); of these latter genes, loss-of-function mutations in PTEN are the most frequent somatic mutations of the signaling pathway, occurring in about 10% of melanomas and being associated with increased AKT signaling (reviewed in References [37,39,82]).

## 4. The MAPK Pathway Mutational Landscape

### 4.1. RAS Mutations

*RAS* gene mutations were firstly associated with melanoma in 1984 [83]; this finding represented the first evidence of the association of an oncogene with this tumor. The most common *NRAS* mutation in melanoma (occurring in more than 80% of *NRAS* mutated samples), is a substitution of glutamine with arginine or lysine at position p.61 (NRAS^Q61R/K/L^) as a result of the c.181C > A transversion (38%), of the c.182A > G transition (34%), or of the c.182A > T transversion (10%) in exon 3 of the gene, respectively [84,85]; other less frequent *NRAS^Q61^* mutations are the *NRAS^Q61E/H/P^* as a result of the point mutations c.181C > G, c.183A > T, and c.182A > C, respectively [84,86] (genomic and protein mutations are designated following the recommendations in References [87,88]). Infrequent *NRAS* mutations can occur as substitutions at positions 12 and 13 of the protein as a result of point mutations in c.34, c.35, c.37, and c.38 positions of exon 2 of the gene (i.e., *NRAS^G12C/R/S/A/D/V^* and *NRAS^G13R/C/A/D/V^* as a result of c.34G > T/C/A, c.35G > C/A/T, c.37G > C/T, and c.38G > C/A/T, respectively) [84,86]; notably, the *NRAS^G12V^*mutation boosts wild-type BRAF (^WT^BRAF) kinase activity 95-fold [89]. While *NRAS^Q61^* mutants lock the GTPase enzymatic task in its active conformation [90], *NRAS^G12^* and *NRAS^G13^* mutations are activating the GTPase by decreasing *NRAS* sensitivity to GTPase activating proteins (GAPs) through alteration of its P-loop [55,91]. 

Mutations of *NRAS*, *KRAS,* and *HRAS* occur in 10–25%, 2%, and 1% of non-uveal melanoma, respectively, including those arising both on sun-exposed and sun-unexposed skin, mucosal, and acral melanomas [92]; in contrast, *RAS* mutations are rarely observed in uveal melanomas [93].

According to the histologic subtype (Table 1), *NRAS* mutations are present in 31% of NMs, in 21% of superficial spreading melanomas (SSMs), in 8% of acral lentiginous melanomas (ALMs), and in 19% of *Lentigo Malignas* (*LMs)* [94]. *NRAS*-mutated melanomas are significantly thicker than wild-type tumors; accordingly, a significant association between *NRAS* mutation and higher Clark’s level of invasion has been found [95,96], suggesting a putative role of this mutation as an adverse prognostic factor; however, the percentage of ulcerated melanomas is low among *NRAS*-mutated samples [97,98]. Hereditary melanomas have a frequency of *NRAS* mutations that is double in comparison with sporadic cases, and the *NRAS^Q61^* mutation has been observed in 95% of primary melanomas from Swedish patients harboring germline *CDKN2A* mutations [99]. 

### 4.2. BRAF Mutations

Melanoma is the second most frequent tumor in which *BRAF* mutations are observed (60% of cases), preceded by hairy cell leukemia (100%), and followed by papillary thyroid cancer (40–60%); *BRAF* mutations have also been identified, albeit at a lower percentage, in astrocytomas (10–15%), colorectal cancer (5–10%), non-small-cell lung carcinoma (3–5%), and other clonal diseases, such as Langerhans and non-Langerhans cell histiocytosis [100,101]; based on this broad occurrence, *BRAF* mutations represent a critical therapeutic target in cancer therapy. 

In melanoma, *BRAF* mutation frequency varies on the basis of the histologic subtype, the anatomical location of the tumor, and the sun exposure pattern. More specifically, more than half of SSMs are *BRAF*-mutated, as well as 43% of NMs, whereas the percentage drops to 15% and 14% in ALM and LMM, respectively (Table 2) [94]; *BRAF*-mutated melanomas are more frequently located in the trunk, and have a significantly higher mutation rate in body sites with intermittent sun exposure (48% vs. 21%) [102]. A correlation between BRAF mutation, higher Breslow thickness, and higher Clark’s level has been quite disputed [103,104,105].

Ethnic and geographical differences have also been reported—in a cohort of Black Africans, in which the incidence of melanoma is low and not related to the sun exposure, the prevalence of *BRAF* mutations was only 8% [106]. Similarly, a low frequency of *BRAF* mutations (19.4%) has been reported in a small cohort of Korean patients [107], probably due to the high prevalence of the ALM subtype in Asia. 

In melanoma, more than 20 *BRAF* mutations have been described; among them, the *BRAF^V600E^* mutation is the most prevalent, accounting for 80–90% of all *BRAF* mutations in melanomas [84,108,109] and being present in almost 60% of all cutaneous melanomas [110]. Notably, *BRAF* mutations are quite infrequent in mucosal melanomas (5% of cases, compared to 15% of NRAS and 26% of c-KIT mutations) [111,112]. 

The p.V600E mutation results in an amino acid substitution at position 600 in the BRAF protein, from a valine (V) to a glutamic acid (E), as a result of the transversion c.1799T > A in exon 15 [113]; this mutation increases the BRAF kinase activity by about 700-fold over ^WT^BRAF, and more than 500-fold over the activated ^WT^BRAF [89]. Other mutations observed in melanomas are the p.V600K (with a prevalence of 7.7%), the p.V600R (1%), the p.V600M (0.3%), and the p.V600D (0.1%) [84], which are all resulting in high activity mutants [89]. The T > A mutation occurring in *BRAF^V600E^* as well as the mutations in *BRAF^V600K/R/D^* are not typical UV-like base changes, nor do they occur at a di-pyrimidine site; however, there is strong evidence that they could still arise from error-prone replication of UV-damaged DNA, possibly as a result of multiple acute episodes of sun exposure [114]. 

The BRAF kinase domain has the prototypical structure of other kinases, including serine/threonine and tyrosine kinases, with a bilobal structure, composed of a small (N-terminal) lobe and a large (C-terminal) lobe separated by a catalytic cleft. In the inactive conformation, as for other kinases, the conserved DFG motif in the activation segment moves toward the P-loop of the small lobe (DFG-out), where the interaction is stabilized by hydrophobic bonds between the glycine-rich segments G596-V600 of the activation segment and the P-loop G464-V471 residues; this conformation makes the catalytic cleft inaccessible to the ATP [89]. Phosphorylation of the activation segment destabilizes the hydrophobic interactions with the P-loop; as a result of that, the activation segment moves toward the large lobe, thus allowing access of the ATP to the catalytic cleft (DFG-in) [89]. Most of the BRAF activating mutations are clustered in two regions of the molecule: (i) the activation domain near the DFG motif (where the *BRAF^V600^* mutations occur) and (ii) the P-loop [115]. Of note, activating *BRAF* mutations are usually mutually exclusive with other melanoma driver mutations, especially *NRAS* mutations [116,117]; interestingly, patients with *BRAF^V600^*-mutated melanomas are significantly younger at diagnosis than those carrying *NRAS*-mutated melanomas [105], while *BRAF^Non-V600^*mutations were associated with older age [118].

When compared to *NRAS^Q61R^* clones, melanoma cells bearing the *BRAF^V600E^* mutation have increased growth rate in soft agar, but lower proliferative ability in liquid medium and after grafting into nude mice [116]. Notably, while the presence of *BRAF/NRAS* mutations in primary tumors do not negatively impact progression free or overall survival, for metastatic lesions, the presence of *BRAF/NRAS* mutations is associated with a shortened survival [119]. 

### 4.3. MEK Mutations

Among MAPKKs (MAP2Ks), somatic mutations have been reported mainly for MEK1 whose overall mutational prevalence in malignant melanomas is 6–7% [84]; the prevalence of these mutations in different melanoma subtypes and anatomical regions is currently unknown. Most of the reported *MEK1* mutations involve exon 3 of the gene with the c.370C > T transition, which results in the gain-of-function mutation p.P124S [120]. Other mutations have been reported in the same exon (c.332T > G (p.I111S), c.362G > C (p.C121S), 371C > T (p.P124L)), as well as in other exons, including exon 2 (c.157T > C (p.F53L)), exon 6 (c.607G > A (p.E203K)), exon 7 (c.790C > T (p.P264S)), and exon 11 (c.1144A > C (p.N382H)) [92,121]. The functional impact of these mutations on the activation of the MAPK pathway is unknown. Interestingly, most of these somatic mutations have also been identified as germline mutations in the cardio-facial-cutaneous (CFC) syndrome, a genetic disorder caused by the aberrant activation of the MAPK pathway during development. Both melanoma and CFC mutations occur in two regions of the MEK protein—in the N-terminal negative regulatory region or in the ATP-binding region of the N-terminal lobe [122].

Notably, MEK1 mutations are often associated with either *BRAF* or *NRAS* mutations [120,121,123]; however, the combinatorial effect of that association on the pathogenesis and treatment of melanoma has been poorly investigated. A general consensus in the field postulates that MEK1 mutations in *BRAF^V600E^* melanomas are linked to both intrinsic and acquired resistance to BRAF inhibitors [124,125,126,127], although these studies were disputed by other evidence [123]. 

### 4.4. ERK Mutations

*ERK* mutations in melanoma are quite infrequent—while the *MAPK1* gene mutations are observed in less than 1% of melanomas (mostly desmoplastic melanomas), *MAPK2* mutations were never scored in this tumor [84]; at odds with melanoma, the gain-of-function *MAPK2^E322K^* mutation is highly represented in a variety of solid tumors, including squamous cell carcinomas of the cervix and of the head and neck [128,129]. In melanoma, most of the *MAPK1* mutations cluster in the ATP-binding domain or, as for the *MAPK2^E322K^*, in the DUSPs-binding domain where the MAPK phosphatases (also known as dual-specificity phosphatases or DUSPs) bind to exert their dephosphorylating activity on ERKs. Several questions about ERK mutations remain open, including the impact of ERK1 mutations on MAPK pathway activation, their prevalence in specific anatomical locations, as well as their occurrence in primary vs. secondary lesions or in primary vs. acquired drug resistance. 

## 5. BRAF Kinase Inhibitors in the Treatment of Melanoma

BRAF kinase inhibitors are small molecules, which can be classified into two types, based on the functional conformation assumed by the BRAF protein when bound to the inhibitor—type-I inhibitors (e.g., vemurafenib, dabrafenib, and encorafenib) stabilize the kinase in its active conformation (DFG-in) by occupying the ATP binding pocket; in contrast, type-II BRAF inhibitors stabilize the kinase in its inactive conformation (DFG-out) by binding to a hydrophobic site that is adjacent to the ATP binding pocket and that is created by the unique conformation assumed by the activation loop in the DFG-out conformation [133]. This key difference in the stabilization of the kinase domain has significant consequences in the inhibitory action.

It has been about 20 years since it was observed that BRAF inhibitors could activate RAF kinases [134]. The mechanism of this paradoxical effect has been partially characterized in recent years—the association of BRAF with the inhibitor promotes the formation of RAF homo/heterodimers (reviewed in Reference [135]). RAF dimerization triggers a pattern of specific autophosphorylation events, which results in a robust potentiation of the kinase activity of the complex by transactivation between protomers [136]. Notably, BRAF/CRAF heterodimers appear to be more active in phosphorylating MEK substrates than BRAF or CRAF homodimers [137]. 

While BRAF inhibitors of both classes stimulate the formation of BRAF-CRAF dimers [89,138], type-II inhibitors are considerably less efficient than type-I inhibitors in stimulating the phosphorylation of downstream targets, i.e., of MEK kinases and, henceforth, in activating the MAPK pathway. Among the possible explanations of this phenomenon, the one that is getting progressively increasing credit is that BRAF molecules locked in the DFG-out conformation by type-II inhibitors have poor efficiency in transactivating the kinase activity of the partner protomer [48,135,138,139]. Interestingly, type-II kinase inhibitors are directed toward the most divergent regions among the various kinases, thus having the additional potential advantage of exerting a highly selective, target-specific action. Based on previous considerations, type-II inhibitors should have greater potential than type-I inhibitors in maximizing BRAF inhibition as single agents or in combined therapies for melanoma treatment. Unfortunately, first generation type-II inhibitors did not hold up to the promises, at least for melanoma treatment. 

Sorafenib, a type-II pan-RAF as well as VEGFR-2/PDGFR-beta kinases inhibitor, was approved for the treatment of unresectable hepatocellular carcinoma, advanced renal cell carcinoma, and thyroid carcinoma refractory to radioactive iodine, but not for melanoma, although 31 clinical trials have been conducted on this tumor (source: https://clinicaltrials.gov/) (accessed on 1 March 2019). The type-II pan-RAF Inhibitor LY3009120 exhibited superior in vitro efficacy in patient-derived melanoma cell lines [140] without paradoxical MEK activation [141], but it did not complete its first clinical trial for melanoma (NCT02014116), due to the lack of sufficient clinical efficacy. The type-II BRAF/EGFR Inhibitor Lifirafenib (BGB-283) demonstrated selective cytotoxicity and inhibition of proliferation of cancer cells harboring *BRAF^V600E^* and *EGFR* mutation/amplification together with in vivo tumor growth inhibition accompanied by partial and complete tumor regressions in both cell line-derived and primary human colorectal tumor xenografts bearing *BRAF^V600E^* mutation. However, clinical trials of this small molecule were not extended to melanoma patients (source: https://clinicaltrials.gov/ct2/show/NCT02610361 and https://clinicaltrials.gov/ct2/show/NCT03641586) (accessed on 1 March 2019).

Second-generation, type-II kinase inhibitors seem to have an increased affinity of the head groups for substrates without paradoxical kinase activation in vitro; however, their efficacy has not been tested in preclinical studies yet. For these reasons, we will mainly focus onto first generation small molecules, with a brief survey of newer type-II kinase inhibitors at the end of the chapter.

### 5.1. Vemurafenib

The identification of *BRAF^V600^* mutations prompted the search for a highly specific kinase inhibitor of the constitutively active kinase with therapeutic value. A high-throughput screening of a library of 20,000 compounds ranging from 150 to 350 daltons identified 238 kinase inhibitors, which were then further refined through protein-inhibitor co-crystallography studies [142]. The optimal high-affinity chemical structure for BRAF kinase inactivation was identified in the 7-azaindole group [142], which was stabilizing the bound protomer in the DFG-in conformation, with the activation loop locked away from the ATP-binding site [143]. Further characterizations identified the first candidate in the pyrrolopyridine N-(3-(5-chloro-1H-pyrrolo[2,3-b]pyridine-3-carbonyl)-2,4-difluorophenyl)propane-1-sulfonamide, also known as PLX4720 (PubChem CID: 24180719). This compound had a high specificity in inhibiting the kinase activity of the *BRAF^V600E^* mutant protein both in vitro and in vivo, displaying excellent oral bioavailability, with an IC_50_ of 13 nM [142]. In a conjunctival melanoma cell line, PLX4720 demonstrated a dose-dependent reduction in downstream MAPK pathway activation, resulting in cell cycle arrest and increased apoptosis [144]. Moreover, the combination of PLX4720 with VEGFR kinase inhibitors induced apoptosis in vitro and tumor regression in animal models, as a result of the synergistic interaction against multiple oncogenic tyrosine kinases [145]. However, treatment of cancer cell lines with PLX4720 showed a potent paradoxical activation of the MAPK pathways [146]. The concomitant identification of PLX4032 (later renamed vemurafenib) as a candidate inhibitor of the same class endowed with superior pharmacokinetics in higher mammals (beagle dogs and cynomolgus monkeys) [143] steered the clinical interest away from PLX4720, which never entered in clinical trials.

Vemurafenib (PLX4032, PubChem CID: 42611257) is a type-I competitive, low molecular weight, serine/threonine kinase inhibitor that functions by binding to the ATP-binding domain of the V600E, V600D, and V600R BRAF mutants. This small molecule was approved for treatment of BRAF-mutated metastatic melanoma by the FDA in July 2011 and by EMA in February 2012, based on the results of the pivotal study BRIM-3 (NCT01006980) [147].

In preclinical studies, it was demonstrated that PLX4032 selectively inhibited the kinase activity of *BRAF^V600^* mutant melanoma cell lines at nanomolar concentrations, blocking cellular proliferation and inducing apoptosis [142,148]; raising its concentration above 100 nM, a number of kinases were progressively inhibited at IC_50_ values, including ^WT^BRAF [143]. Moreover, orally administered PLX4720 was able to inhibit tumor growth in melanoma xenograft models, while, at a higher dosage, it induced tumor regression, without evident toxicity [148,149].

In a clinical context, the dose-escalation phase-I trial BRIM-1 (NCT00405587) [150] established 960 mg twice daily as the maximum tolerable dose [150]. After oral administration, vemurafenib has a median time to maximum drug concentration (T_max_) of about 4 h. It is almost exclusively bound (99%) to albumin and α-1 acid glycoprotein in plasma [151], where it reaches a steady-state concentration after approximately 15–21 days [152]. Vemurafenib has a very limited distribution in the central nervous system due to its dual action as both a substrate and an inhibitor of the drug efflux transporters P-glycoprotein (P-gp) and the breast cancer resistance protein (BCRP) at the blood–brain barrier [153]; since these transporters have a widespread distribution, caution has to be exerted in administering vemurafenib together with other drugs (e.g., digoxin) that are acting as P-gp and/or BCRP substrates in order to avoid suboptimal concentrations of vemurafenib and/or the concomitant drug(s) [152]. Vemurafenib’s half-life is approximately 57 h; its elimination is predominantly hepatic via bile into feces, with a minor role for renal excretion (Table 3). In the liver, vemurafenib is mainly metabolized by cytochrome P450 (CYP) 3A4, which is responsible for the formation of hydroxylated metabolite species (Table 3) [154]; the concomitant generation of glucuronidated and glucosylated metabolites in the liver has been described but not further characterized (Table 3) [152]. A number of clinical studies have observed that the efficacy of drugs metabolized by CYP3A4 (e.g., contraceptive pills) can be considerably reduced when co-administered with vemurafenib (reviewed in Reference [152]). Notably, in mild to moderate hepatic impairment, no alteration of vemurafenib clearance has been observed, thus indicating that no dose reduction is required in this clinical condition [152].

Vemurafenib, administered according to the standard schedule, demonstrated a RR of 53%, with a median duration of response of 6.7 months in the BRIM-2 phase-II trial [155], which enrolled *BRAF^V600E^* mutant patients previously treated for advanced or metastatic melanoma. On this base, therefore, it was designed the BRIM-3, a randomized, active control, open-label, multicenter study comparing vemurafenib monotherapy with dacarbazine (DTIC) [147]. It enrolled 675 patients affected by metastatic melanoma, positive for the mutant *BRAF^V600E^* gene (confirmed by the COBAS test before starting the treatment), who were randomized to receive oral vemurafenib 960 mg twice daily or DTIC administered intravenously (1000 mg/m^2^ on day 1 every 3 weeks). In BRIM-3, the difference in overall survival (OS) between the two treatment arms reached a very high significance, already during the pre-specified interim analysis; therefore, the study was modified to allow DTIC patients to cross-over to receive vemurafenib. At the time of crossover, the study demonstrated a significantly longer median OS for vemurafenib (13.2 months (95% CI: 12.0–15.4)) than for DTIC (9.7 months (95% CI: 7.9–12.8)); a similar finding was also scored for median OS without censoring at crossover (13.6 months for vemurafenib (95% CI: 12.0–15.4) vs. 10.3 months for DTIC (95% CI: 9.1–12.8)) [156]. Despite the short-term benefit in terms of OS, the 2-year survival was 17% in the vemurafenib arm vs. 15.6% of DTIC; notably, the survival benefit of vemurafenib over dacarbazine was more pronounced in patient subgroups with poor prognostic factors (e.g., ≥65 years) and greater disease burden (≥3 metastatic sites), although these data did not reach the statistical significant due to the small patient groups [156]. Good clinical response rates were also demonstrated in both treated (cohort 1) and untreated (cohort 2) patients affected by brain metastases (clinical trial #NCT01253564), where vemurafenib was well-tolerated, without significant nervous system toxicity [157]. Median OS was 8.9 months (range 0.6–34.5; IQR 4.9–17.0) in cohort 1 and 9.6 months (range 0.7–34.3; IQR 4.5–18.4) in cohort 2.

Recently, data from a phase-III, double-blind, randomized, placebo-controlled clinical trial of vemurafenib as adjuvant therapy were published [158]; in that study, named BRIM-8 (NCT01667419), about 500 patients with *BRAF^V600^* mutant melanomas at stage IIC and III at high risk for recurrence were enrolled. In patients with stage IIC, IIIA, or IIIB resected melanomas, the adjuvant treatment with vemurafenib increased the disease-free survival compared to placebo, although the benefit was not statistically significant due to the study design.

Clinical safety of vemurafenib was evaluated in all the 866 patients included in phase-I, phase-II (NCT00949702), and phase-III studies [147,150,159]. The most frequent adverse reactions were skin rash (60%), photosensitivity (37%), development of squamous cell carcinomas and/or keratoacanthoma (21%), arthralgia (18%), nausea (10%), fatigue (8%), and pruritus (6%). Moreover, a statistically significant prolongation of cardiac repolarization was observed, not only in patients treated with vemurafenib, but also in those treated with other tyrosine kinase inhibitors (TKIs), including sorafenib, imatinib, and erlotinib [160]; however, only patients who received vemurafenib were at an increased risk of developing CTCAE (common terminology criteria for adverse events) Grade 3 QTc prolongation (i.e., with an average QTc ≥501ms, a threshold associated with high risk for serious arrhythmias) [160,161].

### 5.2. Dabrafenib

Dabrafenib (GSK2118436, PubChem CID: 44462760), which belongs to the class of sulfanilides, is a type-I kinase inhibitor of the *BRAF^V600E/K/D^*-mutated proteins. It was approved for advanced-stage melanoma treatment by both the FDA and EMA in 2013, on the basis of a multicenter, open-label, phase-III randomized BREAK-3 (NCT01227889) controlled trial [162]. Dabrafenib is a reversible, ATP-competitive kinase inhibitor, with a reported IC_50_ of 0.65, 0.5, and 1.84 nM for *BRAF^V600E^*, *BRAF^V600K^*, and *BRAF^V600D^*, respectively [163]; above 3 nM, dabrafenib reaches the IC_50_ for wild-type RAF members [163], while at > 10 nM, it reaches the IC_50_ for other kinases, including SIK1, NEK11, and LIMK1 [164].

Preclinical studies showed that melanoma cells lines treated with dabrafenib at nanomolar concentration for 72 h displayed a potent inhibition of ERK phosphorylation and cell proliferation [165]. Notably, in RAS-mutated, ^WT^BRAF cancer cell lines, dabrafenib induced robust activation of the MAPK pathway [138,166], possibly through RAF dimerization and/or by relieving inhibitory autophosphorylation of RAF due to its phosphate-binding loop disruption [164]. In a *BRAF^V600E^* mutant xenograft melanoma model, orally administered dabrafenib inhibited ERK activation, downregulated Ki67, and upregulated p27 (cyclin-dependent kinase inhibitor), leading to tumor growth inhibition [167].

Clinical studies have set the recommended dose of dabrafenib at 150 mg orally twice daily, as a single agent or in combination with trametinib [163]; after oral dabrafenib administration, the median time to achieve peak plasma concentration is 2 h with a mean absolute bioavailability of 95% [168]. Dabrafenib is metabolized in the liver, mainly by CYP2C8 and CYP3A4, to form hydroxylated metabolites, which are then further oxidized via CYP3A4 to form carboxy-dabrafenib, subsequently excreted mostly in bile and marginally in urine; the dabrafenib half-life is about 8 h [163].

The phase-I/II trial BREAK-3 [169] randomly assigned 250 patients to receive dabrafenib (150 mg twice daily, orally) or DTIC (1000 mg/m^2^ intravenously every 3 weeks); the median progression-free survival (PFS) was 5.1 months for dabrafenib and 2.7 months for dacarbazine, with a statistically significant difference [169]. Treatment-related adverse events (AEs) occurred in 53% of patients who received dabrafenib and were mainly represented by skin-related toxic effects, fever, fatigue, arthralgia, and headache. This study confirmed the clinical benefit of treatment with dabrafenib, which was previously observed in a noncomparative, phase-I/II trial (NCT00880321)—in this study, dabrafenib 150 mg BID gave a partial response in 24/27 patients after a median treatment period of 7 months and with a median PFS of 5.5 months [170]; these data are in agreement with what was observed for vemurafenib in the previously cited BREAK-3 study, after 2 years of follow-up [156].

Both the NCT00880321 trial and a noncomparative phase-II trial (BREAK-MB; NCT01266967) [171] demonstrate a response to dabrafenib also for brain metastases [171]. BREAK-MB enrolled 172 patients, divided in two cohorts of treated and untreated patients. An overall intracranial response was achieved in 6.7% of treatment-naive and 22.2% of treatment-experienced patients, with significant differences in the median duration of response in relation to the type of mutation. Patients with the V600E mutation reached a median intracranial response of 20.1 months (treatment-naive) and 28.1 months (pretreated), with a median PFS of 16.1 and 16.6 months and a median OS of 33.1 and 31.4 months, respectively. In contrast, the median intracranial duration response was 12.4 (treatment-naive) and 16.6 months (pretreated), in those with V600K mutation, with a PFS of 8.1 and 15.9 months and an OS of 16.3 and 21.9 months, respectively [171]. Also, in this subgroup, the safety profile was acceptable, with AEs similar to those previously observed.

### 5.3. Encorafenib

Encorafenib (LGX818, PubChem CID: 50922675), which belongs to the class of phenylpyrazoles, is a second-generation type-I, ATP-competitive, selective kinase inhibitor, which specifically targets *BRAF^V600E^* and *BRAF^V600k^* [172]. It was approved by the FDA on June 2018 (https://www.fda.gov/Drugs/InformationOnDrugs/ApprovedDrugs/ucm611981.htm) (accessed on 1 March 2019). and by EMA on September 2018 (https://www.ema.europa.eu/en/medicines/human/EPAR/braftovi#authorisation-details-section) (accessed on 1 March 2019). in a combination therapy with the MEK inhibitor binimetinib; based on the approval highlights, the recommended dosage for encorafenib is 450 mg taken orally once a day together with 45 mg of binimetinib taken orally twice daily, approximately 12 h apart. Encorafenib inhibits both wild-type BRAF, CRAF, and the mutated *BRAF^V600E^* with about the same IC_50_ in cell-free assays [173]; in *BRAF^V600E^*-mutant cancer cell lines, encorafenib reaches an IC_50_ at <40 nM, potently inhibiting cell proliferation. An initial dosage of 300 mg was recommended for the phase-II trial NCT01894672, based on the phase-I dose-escalation and -expansion study NCT01436656 [174]. The bioavailability of oral encorafenib is around 85% and it is rapidly absorbed with a T_max_ of 2 h; its half-life is much higher (3.5 h) than that of vemurafenib (0.5 h) and dabrafenib (2 h). These data, together with the very long dissociation half-life of encorafenib from the kinase (>30 h), makes its inhibitory effects last longer than for any other RAF inhibitor [174]. It is metabolized by N-dealkylation in liver microsomes through CYP3A4 as the main contributor (83%), followed by CYP2C19 (16%) and CYP2D6 (1%) (Table 3) [172]; it is equally excreted in urine and feces (Table 3) [172]. Preclinical studies in cell lines and mouse xenografts showed that encorafenib effectively inhibits tumor growth [174]. Also, in BRAF inhibitor-naive patients, encorafenib guaranteed an overall response rate of 60%, with a median PFS of 12.4 months (*n* = 18; 95% CI: 7.4–not reached). However, the ORR and PFS were significantly lower (22% and 1.9 months, respectively) in patients that experienced a previous treatment with another BRAF-inhibitor [175].

At least an adverse effect related to the treatment was always scored in all patients, in both phase-I and phase-II trials. The most commonly reported side effects were arthralgias, nausea, pruritus, palmoplantar hyperkeratosis, and/or erythrodysesthesia; the percentage of squamous cell carcinomas occurring during treatment with encorafenib was significantly lower than with vemurafenib or dabrafenib (2.7%, 21%, and 6.7%, respectively) [175].

### 5.4. Type-II Kinase Inhibitors

The aromatic ether RAF265 (CHIR265, PubChem CID: 11656518) is an orally active, second-generation, type-II pan-RAF kinase inhibitor, which was demonstrated to target both BRAF (wild-type and mutated isoforms) and CRAF in preclinical studies; in addition, it was found to have anti-angiogenic activity through inhibition of the VEGFR-2 kinase [176,177]. The phase-I/II study CHIR-265-MEL01 (NCT00304525) was designed with the aim of determining the safety profile, pharmacokinetics, pharmacodynamics, and maximum tolerated dose of RAF265 in patients with locally advanced and metastatic melanoma [178]. This study showed an objective response rate (ORR) in 12.1% of patients, across all dose levels and independently of the mutational status. In responder patients, the median duration of response was 18.3 months (range, 1.4–51.7 months) despite some significant and prolonged toxicities (mainly thrombocytopenia and toxic retinopathy) [178]. A recent study on lung and pancreatic cancer cell lines have showed that RAF265 induces dimerization of RAF kinases and activation of ERK, which requires concomitant inhibition of MEK to control MAPK pathway paradoxical activation [179].

TAK632 (PubChem CID: 46209401), a 1,3-benzothiazole derivative based on the thiazolo[5,4-b]pyridine class RAF/VEGFR2 inhibitor 1, is another type-II, pan-RAF inhibitor, which recently attracted some interest for a potential antitumoral activity in melanoma. In vitro studies showed a potent dose-dependent antiproliferative effects on both *NRAS*-mutated or *BRAF*-mutated melanoma cells, even with acquired resistance to BRAF inhibitors, with minimal RAF paradoxical activation [180,181]. At present, TAK632 is still in the preclinical phase, whereas another pan-RAF inhibitor of the same class, MLN2480 (formerly known as TAK-580), is in phase-I clinical trial (NCT01425008), evaluating dose escalation and dose expansion in patients with solid tumors, including melanoma; this study was completed on October 2018, but, at the time of writing, no results about this study have been posted.

The benzamide compound AZ628 (PubChem CID: 11676786) is another second-generation, type-II RAF inhibitor that showed a minimal paradoxical MAPK pathway activation in RAF-overexpressing cells [182]. Furthermore, AZ628 inhibited the MAPK signaling more effectively than dabrafenib in a number of carcinoma cell lines co-expressing several different BRAF mutants with CRAF. Similarly, AZ628 plus trametinib had a superior MEK-inhibitory and pro-apoptotic action than dabrafenib plus trametinib, with greater inhibition of cell growth [182], indicating AZ628 as a potential candidate inhibitor for *BRAF*-mutant solid tumors, both in monotherapy or in combination with MEK inhibitors.

### 5.5. Dimeric Compounds and Combinations of BRAF Inhibitors

Studies on BRAF co-crystals with dimer-inducing kinase inhibitors have emphasized the importance of another region of the RAF kinase—the αC-helix region—in the promotion/inhibition of side-to-side dimerization as well as in allosteric transactivation of protomers [183,184,185]. More specifically, the position of the αC-helix stabilized by the kinase inhibitor determines protomer occupancy. An inward orientation of the αC-helix (αC-IN, as promoted by TAK632 and AZ628) favors the side-to-side dimerization and co-occupancy of both protomers by the inhibitor, thus effectively counteracting paradoxical kinase activation [181,184]. Conversely, kinase inhibitors that stabilize the αC-helix in the outward position (αC-OUT, such as vemurafenib) strengthen the formation of hybrid dimers, in which one protomer binds the inhibitor in the αC-OUT conformation, being effectively inhibited, while the second protomer is forced by allosteric interaction in the αC-IN (active) conformation; this latter conformation does not provide access to the kinase inhibitor, thus allowing for paradoxical kinase activation of the second protomer [183,184].

Interestingly, dimeric vemurafenib compounds promote the formation of inactive RAF homodimers as the result of locking both protomers in the αC-OUT conformation; these dimeric compounds have the additional advantage of improved potency and selectivity over monomeric vemurafenib and most of other sulfonide-type kinase inhibitors in vitro [186]. In contrast, dimeric αC-IN compounds (e.g., TAK632) do not promote BRAF dimers formation in vitro, thus resulting in a significantly compromised potency [181].

A system biology-based approach has recently been developed to analyze combinations of different RAF inhibitors by means of a next-generation, mechanistic dynamic model, which was validated by experiments in mutant *NRAS* and *BRAF^V600E^* cancer cells [185]. This predictive model integrates structural elements of the BRAF-inhibitor interaction (i.e., DFG-in/DFG-out and αC-IN/αC-OUT rules), with additional data, including thermodynamics and kinetics of drug-protein interactions, posttranslational modifications, and cell-line mutational status [185]. Kinase inhibitors can be subdivided into three major groups according to the structure assumed by the DFG motif and the αC-helix of the kinase when bound to the inhibitor—(i) αC-IN/DFG-in inhibitors (e.g., SB590885, GDC-0879), (ii) αC-IN/DFG-out inhibitors (e.g., AZ-628 and TAK-632), and (iii) αC-OUT/DFG-in inhibitors (e.g., vemurafenib, dabrafenib, and encorafenib) [185]. According to this prediction model [185], the following combinations of BRAF inhibitors were identified and experimentally proved to be effective in inhibiting ERK kinase activation in vitro according to a specific cancer cell mutational status [185]: (i) The association of αC-IN/DFG-out and αC-OUT/DFG-in inhibitors was most effective in inhibiting the phosphorylation activity of RAF homodimers or RAF homo- and heterodimers in cancer cells bearing the *BRAF^V600E^*/^WT^BRAF or *BRAF^V600E^*/*NRAS^Q61K^* mutations, respectively; (ii) combination of αC-IN/DFG-in and αC-IN/DFG-out inhibitors halted ERK signaling from RAF heterodimers in cancer cells harboring oncogenic *RAS* mutations and ^WT^RAS; (iii) finally, the αC-IN/DFG-out and αC-OUT/DFG-in inhibitors pair was required to extend the inhibition of kinase activity of both RAF homo- and heterodimers in cancer cells bearing both *RAS* and *RAF* activating mutations. Altogether, these studies provide potent rules to establish the best combination of present and future BRAF inhibitors tin order to overcome paradoxical MAPK pathway activation.

## 6. MEK Inhibitors in the Treatment of Melanoma

The discovery of BRAF inhibitors has revolutionized the landscape of melanoma treatment, offering the possibility of far superior response in comparison to that provided by the traditional combination of surgery and chemotherapy. However, responses to BRAF inhibitors are not long lasting due to the acquisition of resistance mechanisms by melanoma cells [150,171]. These mechanisms, extensively reviewed in References [68,187,188], include the acquisition of new genetic mutations [126,189,190] together with epigenetic and/or transcriptomic changes [190]. Anyway, in the majority of advanced melanomas, the central mechanism for the acquired resistance to BRAF inhibitors is the reactivation of the MAPK pathway [68], mainly due to the paradoxical effects previously described. This observation poses the clinical basis for the association of BRAF kinase inhibitors with inhibitors of downstream kinases in the MAPK phosphorylation pathway—i.e., MEK and ERK inhibitors—in order to overcome paradoxical effects by blocking the most important therapeutic “escape route” of the tumor. Furthermore, this combination provides a number of additional advantages, including a more potent and longer lasting clinical response [191,192,193,194], together with a reduced toxicity in comparison with BRAF inhibitor monotherapy.

Besides overcoming paradoxical activation of monotherapy, another key therapeutic indication for MEK inhibitors is for the treatment of *NRAS*-mutant melanomas, which are more aggressive and have a poorer outcome than the *BRAF*-mutant counterparts [96]. In the former subset of tumors, the most promising target therapy to be tested in future clinical trials is a combination of MEK inhibitors (and, possibly, future ERK inhibitors), cell cycle inhibitors (in particular, CDK4/6 inhibitors), and agents inhibiting the PI3K-AKT-mTOR pathway (reviewed in References [85,195,196,197]).

### 6.1. Trametinib

Trametinib (GSK1120212, PubChem CID: 11707110) is a type-III, reversible allosteric, non-ATP-competitive inhibitor of both MEK1 and MEK2; as a type-III compound, it binds within the cleft between the small and large lobes of the kinase, adjacent to the ATP binding pocket, so that both the ATP and the allosteric inhibitor can bind simultaneously to the kinase [48]. Trametinib may be administered as monotherapy, but it is more commonly given in combination with dabrafenib for the treatment of adult patients with unresectable or metastatic melanoma harboring the *BRAF^V600^* mutation [198]. Tramentinb is orally administered as a single dose of 2 mg/day as a single agent or in combination with dabrafenib 150 mg orally taken twice daily; it has a bioavailability of 72%, leading to a peak plasma concentration of 22.2 ng/mL in 1.5 h, as per FDA and EMA labels (https://www.accessdata.fda.gov/drugsatfda_docs/label/2014/204114s001lbl.pdf and https://ec.europa.eu/health/documents/community-register/2017/20170327137432/anx_137432_en.pdf, accessed on 21 March 2019). Trametinib is predominantly metabolized in the liver, via deacetylation alone or with mono-oxygenation and/or glucuronidation; it is not a substrate of the most common CYP450 enzymes, P-gp or BCRP, but inhibits CYP2C8 and induces CYP3A4 in vitro. More than 80% of this compound is excreted in feces, and the remaining metabolites are eliminated in the urine (Table 3).

In the murine melanoma model, trametinib demonstrated a powerful action in controlling tumor growth, despite a high grade of skin toxicity; in contrast, the combination of this MEK inhibitor with the BRAF inhibitor dabrafenib led to a reduction in toxicity, maintaining a high response rate in melanoma treatment [199].

In humans, the effectiveness of monotherapy with trametinib was initially assessed in the NCT00687622 phase-I study, which led to the identification of the 2 mg daily dose as that was associated with the best response and greater safety [200]. In this study, the dose-limiting toxicities were represented by rash, diarrhea, and retinopathy, whereas the most commonly reported AEs were skin rash, diarrhea, edema, fatigue, and nausea; an asymptomatic and reversible reduction in the cardiac ejection fraction and ocular toxic effects were described but infrequently noticed. Objective responses were observed in about 10% of melanoma patients included in this clinical trial, whereas in the NCT01037127 phase-II study, the percentage of responses increased to 25% [201]. In the phase-III METRIC trial (NCT01245062) [202], with PFS as the primary and OS as the secondary endpoint, 322 patients with *BRAF^V600E/K^*-mutated metastatic melanomas were randomly assigned to receive trametinib (2 mg daily), or chemotherapy (intravenous DTIC 1000 mg/m^2^ or paclitaxel 175 mg/m^2^ every 3 weeks) in a 2:1 ratio. Patients enrolled in the chemotherapy group, who showed disease progression, were permitted to crossover to receive trametinib. Monotherapy with this compound was shown to improve both PFS and OS, compared to conventional chemotherapy, ensuring a median PFS of 4.8 months, with a 6-month-rate OS of 81% (vs. 1.5 months and 67% of conventional chemotherapy) [202].

However, the major use of trametinib is in combination with dabrafenib: actually, this combination therapy allows for a significant improvement in clinical responses, with better control of toxicities related to the BRAF inhibitor-induced paradoxical activation of the MAPK pathway. After the initial encouraging results obtained in phase-I/II trials [203], the effectiveness of dabrafenib plus trametinib in advanced melanoma was confirmed in the COMBI-D phase-III trial (NCT01584648) [204], in which 432 previously untreated patients were randomly assigned to receive a combination of dabrafenib and trametinib (150 mg BID plus 2 mg once daily) or dabrafenib and placebo. In patients treated with the combination therapy, both the PFS and OR rate was higher than in the group treated with dabrafenib alone (9.3 vs. 8.8 months and 67% vs. 51%, respectively). Rates of AEs were similar in the two groups, but in the combination treatment group, the incidence of cutaneous squamous cell carcinomas was lower, even if pyrexia was more frequent and severe than with trametinib alone. The comparison with vemurafenib alone also proved the advantages in terms of PFS and OS of the combination therapy, performed in the phase-III COMBI-v clinical trial [193]; this study also confirmed the significantly lower toxicity of the combination therapy compared to vemurafenib. From these positive results, the dabrafenib/trametinib combination was approved for the treatment of metastatic *BRAF^V600E^*^/*K*^-mutated melanomas by FDA in January 2014 and by EMA in August 2013.

In 2015, Long et al. [192] published results from the final overall survival analysis of the COMBI-d, confirming the superiority of the dabrafenib/trametinib combination relative to the number and duration of responses, and progression-free and overall survival. Indeed, the 1-year and 2-year OS rates were 74% and 51%, respectively, in patients treated with the combination therapy vs. 68% and 42% for the group, which received dabrafenib alone. Median OS was 25.1 months (95% CI: 19.2–not reached) in the combination group and 18.7 months in the group who received dabrafenib alone (95% CI: 0.55–0.92; *p* = 0.0107), with a median PFS of 11.0 months (95% CI: 8.0–13.9) in the dabrafenib and trametinib group and of 8.8 months (5.9–9.3) in the dabrafenib group.

This positive outcome has been confirmed also in a long-term follow-up study —patients belonging to the most favorable subgroup in terms of prognosis (i.e., with normal lactate dehydrogenase serum levels and less than three metastatic sites) achieved an OS of 62% if treated with the dabrafenib/trametinib combination [192]. However, regardless of the prognostic factors, the 3-year OS and PFS rates were 44% and 22%, respectively, in patients who received the combination, and 32% and 12%, respectively, in patients treated with the BRAF inhibitor alone [205]. Moreover, a reduction of AEs was confirmed for patients treated with combination therapy, probably due to the reduced paradoxical activation of the MAPK pathway experienced by this latter cohort.

In November 2017, Long et al. [206] published the results from the double-blind, placebo-controlled, phase-III trial COMBI-AD (NCT01682083), which assessed the role of the dabrafenib/trametinib combination therapy on the outcome of patients with resected, stage-III high-risk (lymph node metastasis >1 mm) melanoma with *BRAF^V600E/K^* mutations. All patients had undergone completion lymphadenectomy with no clinical or radiographic evidence of residual regional node disease within 12 weeks before randomization and had recovered from definitive surgery. The 870 patients included in this study were randomly assigned to receive oral dabrafenib plus trametinib (150 mg BID plus 2 mg daily) or two matching placebos for 12 months. After a median follow-up of 2.8 years, the estimated 3-year rate of relapse-free survival was 58% in the treatment group and 39% in the placebo group (95% CI: 0.39–0.58; *p* < 0.001), with a 3-year OS rate of 86% in the treatment group and 77% in the placebo group (95% CI: 0.42–0.79; *p* = 0.0006). Rates of distant metastasis-free survival and freedom from relapse were higher in the combination therapy group than in the placebo group. The safety profile in the adjuvant setting group was similar to that observed in the combination therapy group, without new toxic effects [206].

Recently, due to the possible synergistic effects of BRAF/MEK inhibitors and immune checkpoint blockers, a phase-III trial (COMBI-I, NCT02967692) was designed with the aim to compare the efficacy and safety of a triple combination of dabrafenib, trametinib and the anti-PD1 antibody PDR001 vs. the usual dabrafenib/trametinib combination. The preliminary results obtained in nine patients indicate a manageable safety profile and a promising clinical activity [207]. The completion of this study and the analysis of its results are expected for February 2020. The safety and efficacy of the combination of anti-PD1 antibodies with dabrafenib and trametinib are being evaluated in the NCT02130466 trial, for which the estimated completion date is 2021. Moreover, in July 2015, the randomized phase-III trial NCT02224781, with the aim of evaluating the best treatment sequence in patients with BRAF-mutated advanced melanoma, was started; this study aimed to enroll 300 patients who were randomized to receive dabrafenib and trametinib followed by Ipilimumab and Nivolumab or, vice-versa, Ipilimumab and Nivolumab followed by dabrafenib and trametinib. For this study, the estimated primary completion date has been set for October 2022.

### 6.2. Cobimetinib

Cobimetinib (GDC-0973, PubChem CID: 16222096) is an orally active, reversible, highly selective small-molecule inhibitor for MEK1 and MEK2—its inhibition of the catalytic activity of MEK1 results in reduced phosphorylation and activation of ERK1 and ERK2, with a correlated decrease in tumor cell proliferation in preclinical models. Cobimetinib has been approved for treatment of patients with unresectable or metastatic melanoma with a *BRAF^V600E/K^* mutation, in combination with vemurafenib; its recommended dosage is 60 mg orally taken once daily for the first 21 days of each 28-day cycle until disease progression or unacceptable toxicity is detected (https://www.accessdata.fda.gov/drugsatfda_docs/label/2015/206192s000lbl.pdf). The bioavailability of orally administered cobimetinib is 46% with a half-life of 44 h. Cobimetinib is mainly metabolized via CYP3A oxidation and UGT2B7 glucuronidation, with no major metabolites formed; it is excreted mainly in feces (78%) but also in urine (18%) (Table 3) [208].

The efficacy and safety of cobimetinib in humans were initially evaluated in the BRIM-7 clinical trial (NCT01271803) [209]. Patients enrolled in this study received oral vemurafenib 720 or 960 mg twice daily in combination with various oral cobimetinib regimens; the maximum tolerated dosage, which was selected for use in subsequent studies, was the current recommended dosage, i.e., 60 mg once daily for the first 21 days of each 28-day cycle, and vemurafenib 960 mg twice daily. A confirmed objective response was observed in 87% of BRAF inhibitor naive patients, with a median PFS of 13.7 months (95% CI: 10.1–17.5); also, 15% of patients who had progressed after treatment with vemurafenib alone experienced an objective response, with a median PFS of 2.8 months (95% CI: 2.6–3.4). After an extended follow-up, median OS reached 28.5 months in BRAF inhibitor-naive patients, with a 61% 2-year OS rate [210].

The results of co-BRIM phase-III clinical trial (NCT01689519) led to the FDA approval for the combination therapy in November 2015 [211]. In this study, 495 patients with previously untreated locally advanced or metastatic *BRAF^V600^* mutated melanoma were randomized to receive vemurafenib and cobimetinib or vemurafenib and placebo, with the primary aim to assess the PFS. Both the response rate and the PFS were significantly higher in the combination than in the placebo group (68% vs. 45% and 9.9 vs. 6.2 months, respectively), with a 10% rate of complete response in patients treated with the combination vs. 4% in the control group. The updated efficacy analysis, which was published in 2016 [194], confirmed the clinical benefits of the combination, showing a median overall survival of 22.3 months (95% CI: 20.3–not estimable) for cobimetinib and vemurafenib vs. 17.4 months (95% CI: 15.0–19.8) for placebo and vemurafenib (HR 0.70, 95% CI: 0.55–0.90; *p* = 0.005). No significant differences in high-grade AEs were observed between the two treatment arms (65% vs. 59%), but the number of patients who developed skin cancer was lower among those treated with the combination therapy [212].

At present, further clinical trials are ongoing, with the aim of evaluating the possible synergistic action of the combination therapy with immunological checkpoint inhibitors. The triple combination of vemurafenib, cobimetinib, and atezolizumab (a fully humanized antibody against the protein-programmed cell death- ligand 1 (PD-L1)) was initially studied in a phase-Ib study on *BRAF^V600^* mutant melanoma (NCT01656642) with a manageable safety profile and promising antitumor results (85.3% ORR). In the ongoing phase-III TRILOGY trial (NCT02908672), the triple combination will be compared to vemurafenib/cobimetinib/placebo; the primary completion date for the TRILOGY trial is estimated to be October 2019.

### 6.3. Binimetinib

Binimetinib (MEK162, PubChem CID: 10288191) is an orally available, selective, non-ATP-competitive allosteric inhibitor of MEK1 and 2, which prevents the activation of MEKs-dependent effector proteins and transcription factors, demonstrating a significant antitumor activity both in melanoma cell lines and in murine xenograft models [85].

In pharmacokinetics studies, after the oral administration of 45 mg, at least 50% of the binimetinib dose was absorbed with a T_max_ of 1.6 h; its half-life is 3.5 h, and the route of elimination is mainly fecal (62%) but also urinary (31%). MEK162 activity on melanoma was confirmed by a phase-II clinical study (NCT01320085), which included patients with locally advanced and unresectable or metastatic malignant cutaneous melanoma with *BRAF^V600E^* or *NRAS* mutations; these patients received binimetinib as an oral dose of 45 or 60 mg twice-daily [213]. A partial response was observed in three patients with *NRAS*-mutant (10%) and in two patients with *BRAF*-mutant melanomas (5%), all treated with the 60 mg twice-daily dose, with a median PFS of 4 months [213]. Afterward, the phase-III NEMO trial (NCT01763164) evaluated the clinical effect of binimetinib on advanced *NRAS*-mutated melanomas, in comparison with dacarbazine [175]. This study enrolled 402 patients, previously untreated or progressed after immunotherapy. Binimetinib treatment resulted in higher median PFS compared to dacarbazine (2.8 months; 95% CI: 2.8–3.6 vs. 1.5 months; 95% CI: 1.5–1.7, respectively), even if the interim analysis did not show a significant improvement of the OS.

A more potent and durable inhibition of the MAPK pathway was obtained by combining binimetinib and encorafenib. In the COLUMBUS study (NCT01909453) [214], 577 patients affected by locally advanced, unresectable, or metastatic cutaneous melanoma harboring the *BRAF^V600E^* or *BRAF^V600K^* mutation, were randomly assigned to receive encorafenib monotherapy (300 mg once daily), vemurafenib monotherapy (960 mg twice daily) or encorafenib plus binimetinib (450 mg once daily plus 45 mg twice daily). The combination of encorafenib and binimetinib granted a median OS of 33.6 months (95% CI: 24.4–39.2) in comparison with the OS of 23.5 months (95% CI: 19.6–33.6) obtained with encorafenib alone or with the 16.9 months (14.0–24.5) obtained with vemurafenib (95% CI: 0.47–0.79). Moreover, the combined treatment of encorafenib and binimetinib was better tolerated, with a lower incidence of grade-3 or -4 AEs and a lower rate of drug discontinuation in comparison with encorafenib and vemurafenib alone.

To date, no clinical studies have been designed to evaluate the efficacy and safety of the encorafenib/binimetinib combination in patients with brain metastases or in the adjuvant setting. Recently, the LOGIC-2 trial (NCT02159066) has been devised to investigate the combination of encorafenib and binimetinib with other classes of targeted drugs not yet used in melanoma treatment, such as inhibitors for CDK4/6, FGFR, c-Met, and PI3K. Completion of this study is expected by the end of March 2019. Moreover, in October 2018, the randomized comparative phase-II EBIN study (NCT03235245) began to recruit patients. Its objective is to prospectively evaluate the impact on the PFS of a sequential approach with encorafenib plus binimetinib administered for 12 weeks and followed by combination immunotherapy with Nivolumab plus Ipilimumab. For this study, the estimated primary completion date is April 2022. Relevant data are also expected by the SECOMBIT trial (NCT02631447), a phase-II study evaluating the best sequencing approach with the combination of target agents (encorafenib plus binimetinib) with immunomodulatory antibodies (Ipilimumab plus Nivolumab) in patients with metastatic melanoma bearing *BRAF^V600^* mutations. At the time of writing, SECOBIT trial is ongoing; its estimated primary completion date is April 2021.

In sum, the combination of BRAF and MEK inhibitors has become the state-of-the-art treatment for *BRAF^V600^*-mutant melanomas (i.e., for more than half of all advanced melanomas) and three of these combinations have been approved so far—(i) dabrafenib and trametinib, (ii) vemurafenib and cobimetanib, and (iii) encorafenib and binimetinib. Although, at present, there does not seem to be a difference in the efficacy among these combination therapies, and encorafenib–binimetinib seems to have the best toxicity profile [214]. On the other hand, binimetinib (as monotherapy or in combination therapy) is considered to be the MEK inhibitor of choice for *NRAS* mutant melanomas (reviewed in References [215,216]).

### 6.4. Selumetinib

Selumetinib (AZD6244, ARRY-142886, PubChem CID: 10127622) is a highly selective, ATP-uncompetitive allosteric inhibitor of MEK1/2 [217]. Early preclinical data showed that this compound inhibited growth and survival of melanoma cells in vitro, especially in the presence of a co-inhibition of the PI3K/AKT pathway [218]. Notably, uveal melanomas are rarely mutated in the *BRAF* or *NRAS* genes, but they rather display mutations of two highly homologous genes encoding for the G-protein alpha-subunit q and 11, i.e., *GNAQ* (Gαq) and *GNA11* (Gα11), in about 85% of the patients [219]. As a result of those mutations, uveal melanomas have the constitutive activation of signaling pathways downstream of Gαs, including the MAPK and PI3K/AKT/mTOR pathways [219]. Accordingly, selumetinib alone or in combination with PI3K/AKT/mTOR inhibitors resulted in a significant induction of apoptosis in uveal melanoma cell lines and of melanoma metastasis in a mouse model of hepatic metastasis [218,220,221,222]. Based on these preclinical data, uveal melanomas were chosen as ideal candidate tumors to test the clinical efficacy of selumetinib monotherapy or in combination with PI3K/AKT inhibitors, since direct inhibition of the GNAQ/GNA11 signaling is not possible for its high systemic toxicity. The clinical efficacy of selumetinib was firstly assessed in the NCT00866177 phase-II clinical trial in which 167 patients with stage III/IV, V600E or V600K *BRAF*-mutant, or *NRAS*-mutant melanomas were recruited; among 15 patients who completed the study, one experienced a complete remission and eight a stable disease state, with a progression of the disease in the remaining six patients.

Then, a number of clinical trials were specifically devoted to test selumetinib efficacy in uveal melanomas. In the phase-III, double-blind SUMIT trial (NCT01974752), 152 patients affected by metastatic uveal melanoma but naive from prior systemic therapies, were randomly assigned (3:1) to selumetinib (75 mg twice daily) plus DTIC (1000 mg/m^2^ intravenously on day 1 of every 21-day cycle) or placebo plus DTIC [223]. The primary endpoint of this study was PFS, with OS and ORR as secondary endpoints; the PFS was 2.8 months in patients who received selumetinib plus DTIC, vs. 1.8 months in those patients who were treated with DTIC alone, without statistically significant differences. The ORR was 3% in the first group, whereas no responses were observed in the DTIC-plus-placebo group, with a tolerable safety profile in all patients [223]. Another phase-II trial (NCT01143402) was conceived to compare temozolomide with selumetinib for the progression-free survival (PFS) of patients with melanoma of the eye. This study had the secondary objectives of determining the PFS, OS, and overall RR of AZD6244 and of correlating these parameters with *GNAQ* (Gαq) and *GNA11* (Gα11) mutational status. PFS of the first arm with Temozolomide was 7 (4.3 to 8.4) vs. 15.9 (8.4 to 21.1) of the second arm with selumetinib; for the secondary objectives, only data for the OS were reported showing a tendency of selumetinib to grant a longer survival.

Finally, an intermittent administration protocol of selumetinib in uveal melanoma patients is under evaluation in the multi-center, phase-Ib, NCT02768766 trial; this study is still in the recruiting phase, with an estimated study completion date for September 2019.

### 6.5. Novel MEK Inhibitors

The MEK1/2 inhibitor GDC-0623 (PubChem CID: 42642654) is a potent, ATP-uncompetitive inhibitor of MEK, showing antiproliferative activity in both *KRAS^G13D^* and *BRAF^V600E^*-mutated cancer cell lines; consistently, GDC-0623 showed potent antitumor activity in xenograft models with *KRAS-*mutated cancer stem cells [224]. The NCT01106599 phase-I clinical trial was planned to test the safety, tolerability, and pharmacokinetics of the compound in patients with locally advanced or metastatic solid tumors, including melanomas; this study was completed in August 2014. Preliminary results provide evidence for clinical efficacy, with reasonable tolerability [225].

PD0325901 (PubChem CID: 9826528) is an orally bioavailable, synthetic organic molecule targeting both MEKs. Preclinical studies showed a growth inhibition in xenograft melanoma models, regardless of the BRAF mutational status, due to the inhibition of the MAPK pathway, followed by G1-phase cell cycle arrest and subsequent induction of apoptosis [226]. However, the NCT00147550 pilot trial, which aimed to assess the tolerability and activity of PD0325901 in advanced melanoma and other solid tumors, was terminated before its completion due to an unexpected high incidence of musculoskeletal, neurological, and ocular toxicities [227].

### 6.6. Dual MEK/RAF Inhibitors

The first-in-class, dual MEK/RAF inhibitor RO5126766 (CH5126766, PubChem CID: 16719221) is an allosteric inhibitor with a novel structure based on a coumarin skeleton; it directly binds to MEK1/2, stabilizing a CH5126766-MEK complex in which the MAP2K cannot be phosphorylated by and released from RAF [228]. Preclinical studies showed that RO5126766 effectively inhibited both MEK and ERK phosphorylation in melanoma cell lines with *RAS* or *NRAS* mutations [229] and reduced tumor growth of *KRAS*-mutated carcinoma cell lines in xenograft models [228]. Based on these results, an open-label, phase-I, dose-escalation study (NCT00773526) was conducted, with the aim to assess the recommended phase-II dose, the pharmacokinetics, and pharmacodynamics of the compound. This study enrolled 52 patients, affected by melanoma (*n* = 21) or other advanced or metastatic solid tumors not responding to standard therapies; among them, three partial responses were observed, in two *BRAF*-mutant and one *NRAS*-mutant melanoma patients. The recommended dose for phase-II trials was set to 2.7 mg, with a 4 days on/3 days off schedule. Almost all patients (94.2%) experienced cutaneous AEs, more commonly represented by skin rash; elevated CPK (55.8%), diarrhea (51.8%), and blurred vision (51.9%) were also frequently reported [230]. At present, a phase-I trial (NCT02407509) evaluating the safety of RO5126766 alone and in combination with everolimus (a serine/threonine kinase inhibitor and mTOR inhibitor immunosuppressant, PubChem CID: 6442177) on patients with *BRAF-*, *KRAS*-, and/or *NRAS*-mutant solid tumors or multiple myeloma is ongoing; the estimated primary completion date is for June 2020.

## 7. ERK Inhibitors in the Treatment of Melanoma

Clinical responses to BRAF and MEK inhibitors are transitory due to the rapid development of acquired resistance, hindering the durable efficacy of monotherapies and combination therapies [59]. The search for mechanisms that determine acquired resistance to MAP3K and MAP2K inhibitors has led to the identification of MAPK pathway reactivation as the leading process to sustain tumor growth and spread under kinase inhibition therapy (reviewed in References [75,231,232,233]). This pathway reactivation is due to a variety of genetic and epigenetic events, including mutations in *NRAS* and/or *MAPK* components, and overexpression of signaling receptors at the cell membrane, such as COT, EGFR, PDGFRβ, IGF1R, or MET (reviewed in References [75,234,235,236]). Pharmacological targeting of all these substrates is unfeasible due to their multiplicity; henceforth, the most logical and, potentially, the ultimate target in MAPK pathway inhibition is to drug the downstream kinases of the pathway, i.e., ERK1/2, from which signaling spreads to cytoplasmic and nuclear effectors [61]. However, the identification of clinically useful ERK1/2 inhibitors is quite recent and subsequent to the development of RAF and MEK inhibitors.

In 2013, SCH772984 (Compound 5, PubChem CID: 24866313) was identified and partially characterized as a type-I/II kinase inhibitor, highly selective for ERK1/2 [237]; it demonstrated potent antitumor activity in xenograft models and inhibited cell proliferation in BRAF and MEK inhibitor-resistant cultures. SCH772984 was also found to have a synergistic antiproliferative action, when combined with vemurafenib [238]. No clinical trials have been conducted on this drug so far, due to its poor pharmacokinetics properties, having high clearance and low permeability, which lead to poor absorption and bioavailability in rodents.

In order to improve the pharmacokinetics of SCH772984, another indazole-pyrrolidine derivative, named MK-8353 (formerly, Compound 7, SCH900353, PubChem CID: 58282870) was developed; it displayed good oral bioavailability across multiple species, with an IC_50_ in the low nM range, and no cross-inhibition of MEK1/2. The pharmacokinetic performances were improved over compound 5 by a careful structure-based drug design in which a 3(S)-thiomethyl substitution of the pyrrolidine core was inserted to retard amide metabolism [239]. Dose, schedule, and activity has been evaluated in the phase-I MK-8353-001 study (NCT01358331), which enrolled 26 patients affected by metastatic melanoma, colorectal cancer, or other solid tumors. MK-8353 was well tolerated up to 400 mg twice daily; a clinical antitumor activity was observed in 3 out the 15 evaluable patients, all carrying *BRAF^V600^*-mutant melanomas [240].

Ulixertinib (BVD-523, PubChem CID: 11719003) is another recently characterized ERK1/2 inhibitor, which showed synergistic anti-proliferative effects when used in combination with BRAF inhibitors in a *BRAF^V600E^* xenograft model [241]. However, this compound downregulates the levels of DUSP6, thus hampering the negative feedback mechanism that tames ERK kinase activity [241]. Ulixertinib safety was evaluated in the phase-I clinical trial NCT01781429, which enrolled both patients with *NRAS*-mutant melanomas and *BRAF*-mutant melanomas which progressed on, or were refractory to, BRAF and/or MEK inhibitors. In these subjects, ulixertinib showed a low risk for QT/QTc prolongation or any other effects on ECG parameters [242]. The second objective of this study was the evaluation of clinical efficacy in treated patients, but these data have not yet been released. At present, the recruitment of patients for the phase-II study NCT03417739 is still active, with a plan to enroll 25 patients. This clinical trial aims to assess the efficacy of ulixertinib in patients with metastatic uveal melanoma; its due time is by the end of August 2021.

Recently, the recruitment of patients for another phase-I study (NCT02457793), evaluating the safety and efficacy of the pyrazole amino-pyrimidine derivative GDC-0994 (ravoxertinib, PubMed CID: 71727581), was completed; this compound was administered in combination with cobimetinib in locally advanced or metastatic solid tumors. Data from this study are not yet available. Preclinical studies reported that GDC-0994 was a selective and potent inhibitor of ERK1/2 with an IC_50_ in the subnanomolar range [243]. Interestingly, ravoxertinib inhibits tumor growth and phosphorylation of ERK targets in nude mice harboring human *KRAS* mutant colorectal tumor implants; pharmacokinetic parameters were good in all species tested [243].

Recently, the phase-I studies NCT02711345 and NCT02857270 were started to investigate two other novel ERK inhibitors, i.e., LTT462 (MedGen UID: 925271) and LY3214996 (PubChem CID: 121408882). For these studies, the expected primary completion dates are July 2019 and January 2020, respectively.

## 8. NRAS Inhibition: New Hope to Drug the Undruggable

Ras oncogenes can initiate cancer in animal models; however, their role in sustaining tumor development and progression has not been fully addressed (reviewed in Reference [244]). There is significant in vitro and in vivo evidence that supports oncogenic *NRAS* as a key target for melanoma: (i) Suppression of oncogenic *NRAS^Q61R^* by siRNA results in reduced migration and invasion of human melanoma cells [245]; (ii) extinction of *NRAS^Q61K^* expression in melanomas of an inducible mouse model resulted in rapid, durable, and complete tumor regression within a few days, supporting the idea that constitutively activated *NRAS* acts as a tumor-maintenance oncogene [246]; (iii) notably, MEK inhibitors treatment (selumetinib plus GSK1120212) was unable to induce tumor regression in *NRAS^Q61K^*-mutant murine melanomas [246] as well as in human xenografts [247].

Initial attempts to target *NRAS* were focused on the inhibition of its membrane association, as a result of a post-translational prenylation; membrane association of RAS is required for activation of downstream effectors [248]. This pharmacological strategy led to the development of many potent peptidomimetic farnesyltransferase inhibitors (FTIs), which showed an excellent efficacy in preclinical models, albeit restricted to tumors bearing the *HRAS* mutation [249,250]. However, KRAS and NRAS, in respect with HRAS, undergo an additional step of geranylgeranylation after farnesylation, which retains their membrane association, thus making FTIs useless on tumors harboring *KRAS* and *NRAS* mutations [72,251]. Recently, the development of new substrates for the farnesyltransferase that prevents the alternative prenylation by geranylgeranyltransferase has successfully mislocalized oncogenic *KRAS* in vitro, raising new hopes for direct druggability of RAS family members [252].

Another recent strategy to target RAS implies the modulation of its phosphorylation/dephosphorylation state. RAS gets phosphorylated by SRC-family kinases on Y32; this phosphorylation inhibits the binding of BRAF while promoting the engagement of GAPs, both actions resulting in the switching off of RAS [253]. PTPN11 encodes for the protein tyrosine phosphatase SHP2 that dephosphorylates Y32, thus activating RAS proteins [254]. Mutations that constitutively activates *PTPN11* play oncogenic roles in melanoma by driving anchorage-independent colony formation and tumor growth [255]. Conversely, silencing the constitutively active *PTPN11^E76K^* expression caused regression of melanomas by apoptosis and senescence in a preclinical model [255]; more importantly, the SHP2 inhibitor SHP099 promoted regression of *NRAS^Q61K^* -mutant melanomas in mice models [255].

In contrast with the inhibitory action of Y32 phosphorylation on RAS activity, S89 phosphorylation by the STK19 serine/threonine kinase promotes NRAS interaction with its downstream effectors, thus triggering the activation of both the MEK-ERK and the PI3K-AKT pathways in human melanocyte cell lines [256]. Consistent with these observations, the target inhibition of STK19 by the small molecule ZT-12-037-01 reduces NRAS phosphorylation at S89 and inhibits the growth of NRAS-driven melanomas [256]. More importantly, ZT-12-037-01 effectively reduces cell proliferation and induces apoptosis of xenografted SK-MEL-2 tumors, prolonging the survival rate of tumor-bearing mice [256]. In sum, the new data on the pharmacological inhibition of RAS interaction with its effectors provide substantial preclinical evidence for exploring the potential antitumoral effects of these novel targeted therapies in future clinical trials.

## 9. Conclusions

For decades, the standard therapy for advanced melanoma was limited to chemotherapy, which had low response rates and minimal impact on survival. About 20 years ago [113], the discovery of BRAF mutations in the majority of melanomas changed the therapeutic perspective for this neoplasia; these oncogenic mutations are constitutively activating a signaling pathway—the MAPK pathway—which is at the root of melanomagenesis, supporting cell growth, survival, and spreading of malignant melanocytes. After this discovery, pharmaceutical research focused on the inhibition of the MAPK pathway via small molecules, initially by targeting the mutant BRAF protein and then its downstream effectors, the MEK kinases. Although monotherapy was clinically effective, the combination therapy of BRAF and MEK inhibitors dramatically changed the response rate and survival of melanoma patients. However, the long-term efficacy of this therapy is limited by the onset of acquired resistance, mainly as reactivation of the MAPK pathway by genetic and epigenetic events. The recent introduction in clinical trials of inhibitors of the most distal kinases in the pathway, i.e., the ERK kinases, is providing new hope in the challenge to overcome MAPK pathway resistance and to achieve a new level of control on melanoma growth and spreading. A further layer of control might arise from adding NRAS inhibition and/or immunotherapy to target T-cell checkpoints, as suggested by recent studies (reviewed in References [257,258]). It is not too unrealistic to think that we are at the verge of a new era in melanoma treatment that may cure or, at least, achieve long-term control of, the deadliest form of skin cancer.

## Figures and Tables

**Table 1:** Percentage of *BRAF* and *RAS* mutant melanomas according to histological type and sun exposure.

Histotype	Intermittent Sun-Exposure		Chronic Sun-Exposure	
	BRAF	NRAS	BRAF	NRAS
Superficial Spreading Melanoma [92,111,112]	56%	15%	50%	30%
Nodular Melanoma [92,130]	31%	24%	15%	41%
Acral Lentiginous Melanoma [92,107,131]	12.5%	17%	/	/
Lentigo Maligna Melanoma [131]	/	/	9–16%	14%
	BRAF		NRAS	
Mucosal Melanoma [132]		5%	15%	

**Table 2:** RAF/MEK/ERK inhibitor developed for melanoma treatment.

Inhibitor	Originator	Target Inhibition	Clinical Phase
*VEMURAFENIB ^(1)^ (PLX4032)*	Plexxicon Genentech	BRAF(V600E)	Approved by FDA (2011) and EMA (2012) [147,150,155,159]
*DABRAFENIB ^(2)^ (GSK2118436)*	GlaxoSmithKline	BRAF(V600E)	Approved by FDA (2013) and EMA (2013) [162,169,171]
*ENCORAFENIB ^(3)^ (LGX818)*	Array BioPharma	BRAF (V600E)	Approved by FDA (2018) and EMA (2018) [175]
*PLX4720*	Plexxicon	BRAF (V600E)	Preclinical [148,149]
*TAK-632*	Takeda	pan-RAF	Preclinical [180,181]
*MLN2480 (TAK-580)*	Takeda Millenium	pan-RAF	Phase I NCT02327169 (completed)
*RAF265*	Novartis	pan-RAF and VEGFR2	Phase I NCT00304525 (completed) ^§^ [178]
*TRAMETINIB ^(4)^ (GSK1120212)*	GlaxoSmithKline	Allosteric, MEK	Approved by FDA (2013) and EMA (2014) [192,201,202,204,205,206]
*COBIMETINIB ^(5)^* *(GDC-0973)*	Exelixis Genentech	Allosteric, MEK	Approved by FDA (2015) and EMA (2015) [209,210,211,212]
*BINIMETINIB ^(6)^* *(MEK162)*	Array BioPharma	Allosteric, MEK	Approved by FDA (2018) and EMA (2018) [213,214]
*SELUMETINIB* *(AZD6244)*	Array BioPharma Astra Zeneca	Allosteric, MEK	Phase III NCT01974752 (SUMIT) and NCT01143402 (completed—has results) [223]
*RO5126766*	Roche Chugai	Dual RAF/MEK	Phase I NCT00773526 (completed) [230]
*GDC-0623*	Genentech	Allosteric, MEK	Phase I NCT01106599 (completed) [225]
*PD0325901*	Pfizer	MEK	Phase I NCT00147550 (prematurely discontinued due to ocular and neurological toxicity) [227]
*SCH772984*	Merck & Co.	Dual mechanism, ERK	Preclinical [238,240]
*MK-8353* *(SCH 900353)*	Merck & Co.	Dual mechanism, ERK	Phase I NCT01358331 (completed) [240]
*ULIXERTINIB (BVD-523)*	BioMed Valley	ERK 1/2	Phase I NCT01781429 (completed) Phase II NCT03417739 (recruiting) [242]
*GDC-0994*	Genentech	ATP-competitive, ERK	Phase I NCT02457793 (completed) [243]
*LTT462* *(CLXH254X2102)*	Novartis	ATP-competitive, ERK	Phase I NCT02711345 (recruiting)
*LY3214996*	Ely-Lilly	ATP-competitive, ERK	Phase I NCT02857270 (recruiting)

Trade name: ^(1)^: Zelboraf; ^(2)^: Tafinlar; ^(3)^: Braftovi; ^(4)^: Mekinist; ^(5)^: Cotellic; ^(6)^: Mektovi. Licensed by: ^(1)^ and ^(5)^: Roche; ^(2)^ and ^(4)^: Novartis; ^(3)^ and ^(6)^: Array BioPharma. ^§^ Phase II portion of study (dose expansion) was cancelled in December 2011.

**Table 3:** Pharmacological characteristics of BRAF/MEK inhibitors.

	Accession Number *	Daily Dose	Peak	Metabolism	Excretion	Half-Life
**Vemurafenb**	DB08881	960 mg BID	3 h	CYP3A4	Feces 94% Urine 1%	57 h
**Dabrafenib**	DB08912	150 mg BID	2 h	CYP2C8 and CYP3A4	Feces 71% Urine 23%	8 h
**Encorafenib**	DB11718	450 mg	2 h	CYP3A4 (83%) CYP2C19 (16%) CYP2D6 (1%)	Feces 47% Urine 47%	3.5 h
**Trametinib**	DB08911	2 mg	1.5 h	Deacetylation and mono-oxygenation	Feces 80% Urine 19%	3.9–4.8 days
**Cobimetinib**	DB05239	60 mg	NA	CYP3A and UGT2B7	Feces 76% Urine 18%	44 h
**Binimetinib**	DB11967	45 mg BID	1.6 h	UGT1A1	Feces 62% Urine 31%	3.5 h
**Selumetinib**	DB11689	NA	NA	NA	NA	NA

* www.drugbank.ca/drugs. (accessed on 1 March 2019).

## Abbreviations

AEs	Adverse Events
ALM	Acral Lentiginous Melanoma
BID	Bis in Die
CI	Confidence Interval
CNS	Central Nervous System
CTCAE	Common Terminology Criteria for Adverse Events
DTIC	Dacarbazine
HR	Hazard Ratio
*LM*	*Lentigo Maligna*
NM	Nodular Melanoma
ORR	Overall Response Rate
OS	Overall Survival
PFS	Progression Free Survival
SSM	Superficial Spreading Melanoma
